# Differential expression of T helper cytokines in the liver during early pregnancy in sheep

**DOI:** 10.21451/1984-3143-AR2018-0141

**Published:** 2019-10-23

**Authors:** Ling Yang, Jiachen Bai, Zimo Zhao, Ning Li, Yujiao Wang, Leying Zhang

**Affiliations:** Department of Animal Science, College of Life Sciences and Food Engineering, Hebei University of Engineering, Handan, China.

**Keywords:** liver, pregnancy, T helper cytokine, sheep

## Abstract

Liver plays important roles in the innate and adaptive immunity, and contributes to the maternal immune adjustments during pregnancy in mice and rats. T helper 1 (Th1) and Th2 cytokines are related to immune response. However, expression of Th1 and Th2 cytokines in maternal livers is unclear during early pregnancy in sheep. In this study, livers were collected on day 16 of the estrous cycle and on days 13, 16 and 25 of pregnancy (n = 6 for each group) in ewes, and qRT-PCR, western blot and immunohistochemistry were used to analyze the expression of Th1 and Th2 cytokines in the livers. Our results showed that interferon-gamma (IFN-γ), interleukin (IL)-2, IL-4, IL-6 and IL-10 were downregulated, and IL-5 was upregulated in the livers during early pregnancy. Furthermore, there was no effect for early pregnancy on expression of TNF-β in the livers, and the IFN-γ protein was limited to the endothelial cells of the proper hepatic arteries and portal veins. In conclusion, early pregnancy exerted its effect on the liver to regulate the Th cytokines expression, but there was no evident shift from Th1 to Th2 cytokines, which may be necessary for the maternal hepatic immune adjustments during early pregnancy in sheep.

## Introduction

There exists maternal immune tolerance that the adaptive immune responses against the fetus and placenta are downregulated (Williams, 2012). During early pregnancy, the conceptus produces cytokines and chemokines, which results in the modulation of maternal immune system in sheep and cattle (Hansen, 2011; Yang *et al*., 2014), and conceptus and progesterone (P4) induce a shift of cytokine-production profile from T helper 1 (Th1) to Th2 in maternal endometrium in ruminants (Hansen, 2011; Zhang et al., 2015). Th1 cytokines, including interleukin (IL)-2, interferon-gamma (IFN-γ) and tumor necrosis factor beta (TNF-β), are implicated in the cellular immune process, proinflammatory responses and autoimmune responses. Th2 cytokines, such as IL-4, IL-5, IL-6 and IL-10, are essential for antibody-mediated immune response (Ott and Gifford, 2010). Th1/Th2 balance is necessary for the maintenance of successful pregnancy, and treatment with Th1 cytokines results in the abortions in normal pregnant mice (Raghupathy *et al*., 2000). There is a Th2 phenomenon in normal pregnancy in cattle (Raghupathy *et al*., 2000), and the patients with reproductive failure are associated with an elevation of Th1/Th2 ratio (Han and Lee, 2018). We previously reported that there were downregulation of IFN-γ and upregulation of IL-4 and IL-10 in peripheral blood mononuclear cells (PBMCs) during early pregnancy in cattle (Yang *et al*., 2018). There were a downregulation of TNF-β and IL-2 and an upregulation of IL-5 and IL-10 in the maternal lymph nodes during early pregnancy in sheep (Yang *et al*., 2019).

Liver has a wide variety of necessary functions, including digestion, metabolism and immunity. Liver plays an important role in the innate and adaptive immunity, and also contributes to the extrathymic T-cell development (Parker and Picut, 2005). P4 causes an upregulation of the asialoglycoprotein receptor and the mannose receptor in the liver during pregnancy in mice (Mi *et al*., 2014), and pregnancy induces a noticeable modulation in the maternal hepatic gene expression, which is related with cell proliferation and cytokine signaling in rats (Bustamante *et al*., 2010). Nuclear factor erythroid 2-related factor 2 is implicated in regulating the maternal hepatic adaptations to the pregnancy through the mammalian target of rapamycin signaling pathway in mice (Zou *et al*., 2013), and there is an increase in the mRNA and activities of cytochrome P450 2D and cytochrome P450 26a1 during pregnancy in mice (Topletz *et al*., 2013). The liver is accurately implicated in maternal immune adjustments in mice and rats. However, it was unclear that early pregnancy exerts effects on expression of Th cytokines in ovine liver. In this study, the expression of IFN-γ, TNF-β, IL-2, IL-4, IL-5, IL-6 and IL-10 was analyzed in the livers from nonpregnant ewes and early pregnant ewes, which may be useful to understand the maternal hepatic immune adjustments during early pregnancy in sheep.

## Materials and Methods

### Animals and experimental design

Healthy multiparous Small-tail Han ewes with similar age (18 ± 2 months) were housed free from drugs and other stress in the Hebei Province, China. All procedures were approved by the Hebei University of Engineering Animal Care and Use Committee, and humane animal care and handling procedures were followed throughout the experiments. Ovine estrous behavior was assessed using vasectomized rams. The ewes of three pregnant groups (days 13, 16 and 25 of pregnancy) were mated twice with fertile rams at a 12-h interval after the detection of estrous behavior, but the nonpregnant ewes (day 16 of the estrus cycle) were mated with a vasectomized ram after the assessment of estrous behavior (day 0). The effects of early pregnancy on the expression of Th cytokines in the ovine livers are mainly due to P4 and IFNT. There were significantly higher concentrations of P4 on days 12-13 in plasma, and lower concentrations of P4 on days 15-16 during the ovine estrous cycle (Mcnatty *et al*., 1973). IFNT (Protein X) and additional proteins were detected between days 14 and 21 in sheep (Godkin *et al*., 1982). Therefore, hepatic samples were sampled at days 13, 16, and 25 of pregnancy, and day 16 of the estrous cycle at the time of slaughter. Pregnancy was confirmed as the presence of a conceptus in the uterus after dissection. Hepatic transverse pieces (0.3 cm^3^) were fixed in fresh 4% (w/v) paraformaldehyde in PBS buffer (pH 7.4), and the remaining portions of hepatic samples were frozen in liquid nitrogen for subsequent quantitative real-time PCR (qRT-PCR) and western blot analysis.

### RNA extraction and qRT-PCR assay

Total RNA extraction of the hepatic samples was performed with TRIzol (Invitrogen, California, USA) according to the manufacturer’s instruction, and approximately 1 μg of the total RNA was reverse transcribed into cDNA using a FastQuant RT kit (Tiangen Biotech Co., Ltd. Beijing). Primers were designed and synthesized by Shanghai Sangon Biotech Co., Ltd. (Table 1), and estimated by BLAST (https://blast.ncbi.nlm.nih.gov/Blast.cgi) at NCBI. Amplification efficiencies of the primer sequences of IFN-γ, TNF-β, IL-2, IL-4, IL-5, IL-6, IL-10 and GAPDH were evaluated before quantification, and a SuperReal PreMix Plus kit (Tiangen Biotech) was used for qRT-PCR, according to the manufacturer’s instruction. The 2^-ΔΔCt^ analysis method (Livak and Schmittgen, 2001) was used to quantify the relative expression values for qRT-PCR assay, with GAPDH as the housekeeping gene to normalize the data, and the mixture of the four groups was used as the mean CT.

**Table 1:** Primers used for qRT-PCR of ovine Th1 and Th2 cytokines

Gene	Primer	Sequence	Size (bp)
IL-2	Forward	AAACCTGAACACCAGAGAGAT	117
	Reverse	GCCTTTACTGTCGCATCA	
IFN-γ	Forward	TTGAACGGCAGCTCTGAGAA	124
	Reverse	TTGGCGACAGGTCATTCATC	
TNF-β	Forward	CCACTGACGGGCTTTACCT	141
	Reverse	TGATGGCAGAGAGGATGTTG	
IL-4	Forward	CCAAAGAACGCAACTGAGAA	120
	Reverse	GCTGCTGAGATTCCTGTCAA	
IL-5	Forward	CATCTGCGTTTGACCTTGG	139
	Reverse	AGTTCCCATCACCTATCAGCA	
IL-6	Forward	CGAGTTTGAGGGAAATCAGG	118
	Reverse	GTCAGTGTGTGTGGCTGGAG	
IL-10	Forward	CTCTGTTGCCTGGTCTTCCT	169
	Reverse	TGTTCAGTTGGTCCTTCATTTG	
GAPDH	Forward	GGGTCATCATCTCTGCACCT	176
	Reverse	GGTCATAAGTCCCTCCACGA	

### Western blot analysis

Hepatic protein samples were prepared by RIPA Lysis Buffer (Biosharp, BL504A), and concentrations of proteins were detected using a BCA Protein Assay kit (Tiangen Biotech). Protein samples were separated by 12% SDS-PAGE, and transferred onto polyvinylidene fluoride membranes (Millipore, Bedford, MA, USA). The membranes were incubated with primary antibodies at 37 ^o^C for 1 h, and the primary antibodies included a mouse anti-IFN-γ monoclonal antibody (Abcam, ab27919, 1:1000), a mouse anti-TNF-β monoclonal antibody (Santa Cruz Biotechnology, Inc., SC-28345, 1:1000), a rabbit anti-IL-2 polyclonal antibody (Abcam, ab193807, 1:1000), a mouse anti-IL-4 monoclonal antibody (Bio-Techne, MAB2468, 1:1000), a mouse anti-IL-5 monoclonal antibody (Santa Cruz Biotechnology, Inc., SC-8433, 1:1000), a rabbit anti-IL-6 polyclonal antibody (Abcam, ab193853, 1:1000) and a mouse anti-IL-10 monoclonal antibody (Santa Cruz Biotechnology, Inc., SC-32815, 1:1000). Secondary goat anti-mouse IgG-HRP (Biosharp, BL001A) and goat anti-rabbit IgG-HRP (Biosharp, BL003A) were diluted to 1:20000 (37^o^C for 40 min). Protein signals were visualized using a Pro-light HRP chemiluminescence detection reagent (Tiangen Biotech). Sample loading was monitored with an anti-GAPDH antibody (Santa Cruz Biotechnology, Inc., sc-20357) at a dilution of 1:1000. Quantity One V452 (Bio-Rad Laboratories) was used to quantify the intensity of the blots, and the relative levels were calculated using GAPDH.

### Immunohistochemistry analysis

The embedded hepatic samples were cut into sections (0.5 μm) and mounted on glass slides. The sections were deparaffinized in xylene and rehydrated in ethanol. The sections were stained by hematoxylin and eosin (HE). Immunohistochemical localization for IFN-γ in the hepatic tissue was performed using the mouse anti-IFN-γ monoclonal antibody (Abcam, ab27919, 1:100). For a negative control, non-immune goat serum was used in place of the primary antibody. A DAB kit (Tiangen Biotech) was used to visualize the antibody binding sites in the tissue sections. Finally, the images were captured using a light microscope (Nikon Eclipse E800, Japan) with a digital camera AxioCam ERc 5s, and the intensity of staining and density of the stained cells were analyzed through the images. The immunostaining intensity of the different hepatic tissue samples from different ewes (n = 6 for each group) was rated by 2 different investigators in a blinded fashion, and histological subtypes were analyzed by assigning an immunoreactive intensity of a scale of 0 to 3, as described in a previous report (Kandil *et al*., 2007). An intensity of 3+ was given to the cells with the highest staining intensity, and an intensity of 0 was assigned to the cells with no immunoreactivity.

### Statistical analyses

Experimental groups consisted of six replicates, and statistical analysis was performed in a completely randomized design using the Proc Mixed models of SAS (Version 9.1; SAS Institute, Cary, NC, USA). P < 0.05 were considered significantly different.

## Results

### Relative expression levels of Th1 cytokines mRNA and proteins in the livers

**Figure 1:**
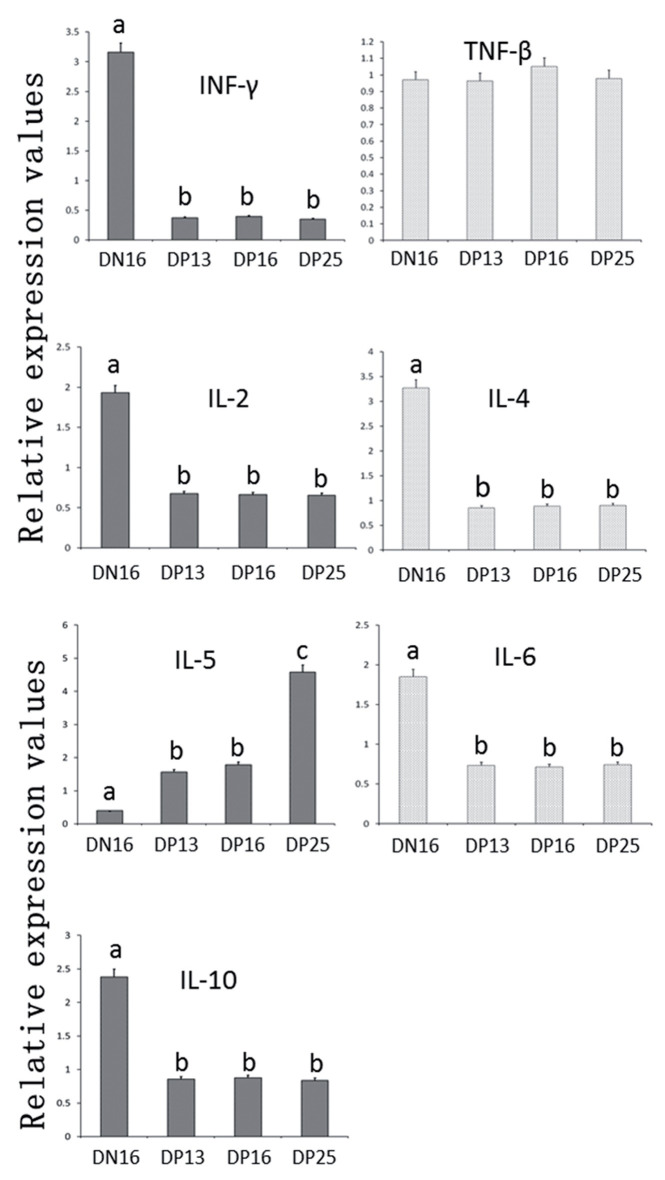
Relative expression values of Th1 cytokines (IL-2, IFN-γ and TNF-β) and Th2 cytokines (IL-4, IL-5, IL-6 and IL-10) mRNA in the livers measured by quantitative real-time PCR in ewes. Note: DN16 = Day 16 of nonpregnancy; DP13 = Day 13 of pregnancy; DP16 = Day 16 of pregnancy; DP25 = Day 25 of pregnancy. Significant differences (P < 0.05) are indicated by different letters within the same color bar.

**Figure 2:**
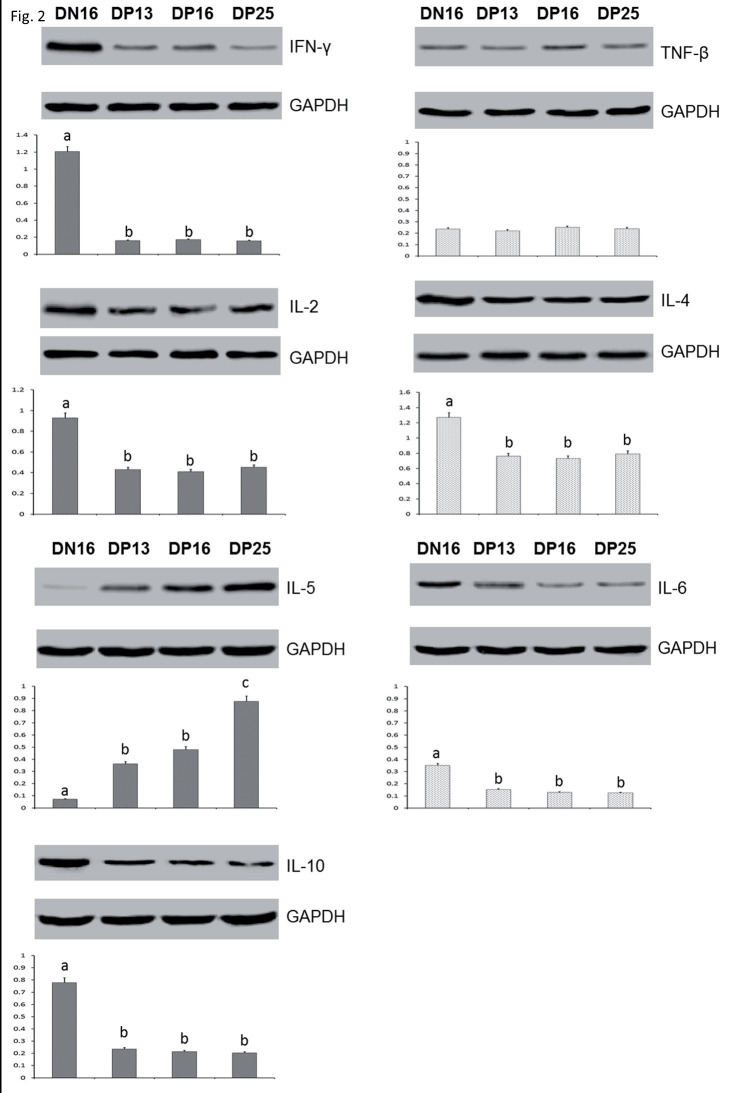
Expression of Th1 cytokines (IL-2, IFN-γ and TNF-β) and Th2 cytokines (IL-4, IL-5, IL-6 and IL-10) proteins in the livers analyzed by western blot in ewes. Note: DN16 = Day 16 of nonpregnancy; DP13 = Day 13 of pregnancy; DP16 = Day 16 of pregnancy; DP25 = Day 25 of pregnancy. Significant differences (P < 0.05) are indicated by different superscript letters within the same color bar.

The qRT-PCR assay and western blot analysis revealed (Fig. 1, Fig. 2) that the expression levels of IFN-γ, IL-2 mRNA and proteins were higher in the liver on day 16 of nonpregnancy than that in the pregnant groups (P < 0.05), but there was no significant difference among the pregnant groups (P > 0.05; Fig. 1, Fig. 2). Furthermore, TNF-β mRNA and protein were expressed in the livers, but there was no significant difference among the four groups in ovine livers (P > 0.05; Fig. 1, Fig. 2).

### Relative expression levels of Th2 cytokines mRNA and proteins in the livers

There was a downregulation of the relative expression levels of IL-4, IL-6, IL-10 mRNA and proteins in the three pregnant groups (P < 0.05), but there was no significant difference among the three pregnant groups (P > 0.05; Fig. 1, Fig. 2). The expression levels of IL-5 mRNA and protein were higher on day 25 of pregnancy than that on day 16 of nonpregnancy, days 13 and 16 of pregnancy (P < 0.05), and the expression levels of IL-5 mRNA and protein were lower on day 16 of nonpregnancy than that in the three pregnant groups (P < 0.05), but there was no significant difference between days 13 and 16 of pregnant ewes (P > 0.05; Fig. 1).

## Discussion

In this study, there was a decrease in the levels of IFN-γ mRNA and protein during early pregnancy (Fig. 1, Fig. 2). As the only member of the type II class of interferon, IFN-γ regulates immunologically relevant genes at a transcriptional level to be implicated in a series of cellular programs (Schroder *et al*., 2004). IFN-γ is a Th1-type cytokine that generally counteracts the Th2 response and increases Th1-adapted immune responses through enhancement of the development of naive T cells into Th1 cells (Nakagome *et al*., 2009). IFN-γ is also the most potent inducer of class II major histocompatibility complex (MHC) genes through three DNA elements in the promoter regions of class II MHC genes (Ting *et al*., 1994). There is a decrease of IFN-γ at the feto-maternal interface during early pregnancy in humans (Saito, 2000), in the endometria on day 7 after oestrus at the correct developmental stage in beef heifers (Beltman *et al*., 2013) and in the bovine PBMCs during early pregnancy (Yang *et al*., 2018). It has been reported that IFN-γ plays a key role in mounting liver environment for the development of hepatic NK cells (Wu *et al*., 2012). Therefore, the downregulation of IFN-γ in the maternal liver during early pregnancy may be necessary for the successful pregnancy in sheep.

TNF-β is a member of the tumor necrosis factor family produced by lymphocytes, is also known as lymphotoxin-alpha (LT-α), and has a powerful effect on the maintenance of the immune system, including the development of secondary lymphoid organs (Ruddle, 2014). As a signaling molecule, TNF-β is implicated in a variety of physiological processes, including the lymphoid tissue development and lymphocyte activation (Bauer *et al*., 2012), and is essential for the host protection against cerebral tuberculosis in the activation of innate immune cells in TNF-deficient mice (Francisco *et al*., 2015). TNF-α, another member of tumor necrosis factor superfamily, is involved in proceeding decidualization, placentation, and prevention from abortion and terminating the fetal life (Ozbilgin *et al*., 2015), but it is reported that TNF-β is associated with an increased risk of preeclampsia and poor fetal growth among European Americans (Harmon *et al*., 2014). In this study, there was no significant difference in the expression of TNF-β mRNA and protein in the livers among the nonpregnant group and early pregnant groups (Fig. 1, Fig. 2), which indicated that TNF-β may be not crucial for the hepatic immune regulation during early pregnancy in sheep.

As a pleiotropic cytokine, IL-2 can drive T-cell growth, augment NK cytolytic activity, and induce the differentiation of regulatory T cells (Liao *et al*., 2011). IL-2 enhances naive CD4^+^ T-cell differentiation into Th1 and Th2 cells, plays key roles in the development and maintenance of T regulatory cells and activation-induced cell death, which are implicated in mediating immune tolerance and limiting inappropriate immune reactions (Liao *et al*., 2013). The IL-2 serum concentrations markedly increased from day 0 to day 10, and gradually decreased from day 10 to day 60 during early pregnancy, but the serum concentrations of IL-2 at the corresponding period of abortion were markedly greater than that on day 60 of pregnancy in goats (Chen *et al*., 2016). Pregnancy rate is decreased in the mouse model of endometriosis, and the level of IL-2 was increased in nonpregnant mice in the endometriosis group, which suggest that the high level of IL-2 may lead to infertility (Bilotas *et al*., 2015). Our results indicated that there was a downregulation of IL-2 mRNA and protein in the livers during early pregnancy (Fig. 1, Fig. 2). IL-2 is one of Th1 cytokines that are generally adverse to pregnancy maintenance. Therefore, the downregulation of IL-2 in the livers may be essential for maintaining a normal pregnancy in sheep.

IL-4 is a potent factor that directs differentiation of naive Th cells (Th0 cells) into Th2 cells (Chen *et al*., 2004), and decreases the production of Th1 cells and IFN-γ (Sokol *et al*., 2008). It has been reported that a shift towards Th1-type immunity (increase in IL-2/IL-4 and IFN-γ/IL-4 ratios) in women with preeclampsia (Molvarec *et al*., 2013). We found that the relative levels of IL-4 mRNA and protein were lower in pregnant groups (Fig. 1, Fig. 2), but the IL-2, IFN-γ mRNA and proteins were also lower in pregnant groups, so there may not exist a shift towards Th1-type immunity in the livers of the pregnant ewes. Furthermore, the level of IL-4 is significantly greater in bitches with pyometra compared with the females in dioestrus, anoestrus and pregnancy (Maciel *et al*., 2014). IL-4 content is higher in the fetal portion of the placenta in rats exposed to air pollution than that in the rats exposed to filtered air before and during pregnancy (de Melo *et al*., 2015). Therefore, the lower level of IL-4 in the livers may be owing to a lower hepatic inflammatory reaction during early pregnancy, which may be helpful for the maternal hepatic immune regulation in sheep.

IL-5 is produced by Th2 cells and mast cells, can stimulate B cell growth and increase immunoglobulin secretion through binding to its receptor. Placental growth factor enhances IL-5 secretion by the CD4^+^ T cell, and inhibits the proliferation of naive CD4^+^ T cell, which are beneficial for the normal pregnancy in humans (Lin *et al*., 2007). The spontaneous *in vitro* secretion of IL-5 in the cell culture supernatants of blood mononuclear cells from normal pregnancy is higher than that from preeclampsia in humans (Jonsson *et al*., 2005). Pregnancy-specific glycoprotein increases the proliferation of IL-5-secreting cells *in vivo*, which protects against *Listeria monocytogenes* infection during pregnancy, is involved in the successful pregnancy in mice (Martínez *et al*., 2012). *Schistosoma japonicum* infection induces a large amount of IL-5 produced by IL-5-producing T cells in mouse liver (Xie *et al*., 2013). Our results showed that the expression of IL-5 mRNA and protein was significantly high in the pregnant groups (Fig. 1 and Fig. 2). Therefore, the upregulation of IL-5 in maternal liver may be induced by the conceptus, and necessary for pregnancy maintenance in sheep.

IL-6 is a multifunctional cytokine, and implicated in the immune adaptation for tolerance in pregnancy (Prins *et al*., 2012). IL-6 participates in embryo-uterine interactions, and is helpful for successful implantation of conceptus during early pregnancy in pigs (Blitek *et al*., 2012). It has been reported that IL-6 and its receptor are upregulated dramatically in the endometrial tissues during mid-to late-pregnancy and decreased at term, but the expression of IL-6 mRNA is lower in the porcine uterine endometrium in pregnant group than that during the estrous cycle on days 12 and 15 postestrus (Yoo *et al*., 2017). In mice, the expression of IL-6 is the highest in the proestrus phase, and is influenced by administration of estrogen and P4 (De *et al*., 1992). IL-6 also induces *in vitro* endometrial synthesis of estrogen in on days 15 to 16 of pregnancy in pigs (Franczak *et al*., 2013). Our data shown that IL-6 mRNA and protein were significantly downregulated in the pregnant groups (Fig. 1, Fig. 2), which indicated that IL-6 may participate in the function of estrogen, and downregulation of IL-6 in the maternal liver may be necessary for the maternal hepatic immune adaptations for tolerance during early pregnancy in sheep.

IL-10 is an anti-inflammatory molecule, and functions as a necessary protective agent to participate in the regulation of maternal immune tolerance and maintenance of normal pregnancy during pregnancy (Cheng and Sharma, 2014). However, this study shown that the levels of IL-10 mRNA and protein in pregnant groups were lower than that in nonpregnant group (Fig. 1 and Fig. 2). It has been reported that IL-10 plays a role in regulating placental development and foetal programming, and IL-10 knockout results in increases in the foetal weight and placental size in IL-10 null mutant mice (Roberts *et al*., 2003). In order to adapt the demands of pregnancy, there exists maternal hepatic growth, including hepatocyte proliferation and size increase in mice (Dai *et al*., 2011; Wang *et al*., 2015). It is supposed that the hepatocyte proliferation and size increase may be related with the decrease in IL-10. Therefore, it was suggested that the downregulation expression of IL-10 in maternal liver may be responded to the demands of pregnancy, and be beneficial for maternal liver to adapt to immune tolerance in sheep.

**Figure 3:**
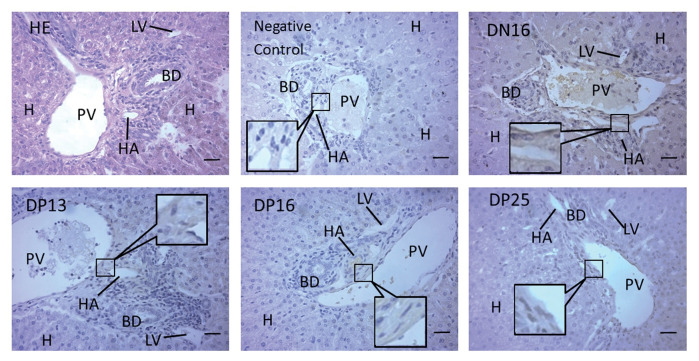
Immunohistochemical localization of IFN-γ protein in the livers. Liver is divided into lobes, and lobe is made up of hepatic lobules. A portal triad is a component of the hepatic lobule, consists of proper hepatic artery (HA), hepatic portal vein (PV), small bile ductile (BD) and lymphatic vessels (LV). Note: HE = stained by hematoxylin and eosin; H = hepatocyte; DN16 = Day 16 of nonpregnancy; DP13 = Day 13 of pregnancy; DP16 = Day 16 of pregnancy; DP25 = Day 25 of pregnancy. Bar = 50 µm.

The liver is divided into lobes, and lobe is made up of hepatic lobules that include hepatocyte plates and a central vein (Frevert *et al*., 2005). A portal triad is a component of the hepatic lobule, consists of proper hepatic artery, portal vein, small bile ductile and lymphatic vessel (Kmieć, 2001). Our immunohistochemistry result shown that the immunostaining for the IFN-γ protein was limited to the endothelial cells of the proper hepatic arteries and portal veins (Fig. 3). The staining intensity for the IFN-γ was higher on day 16 of the estrous cycle than that during early pregnancy (Fig. 3). Blood flows from the portal veins and hepatic arteries entry into the hepatic lobules, and then drain to the central vein (Kmieć, 2001). Therefore, we suggested that the changed expression of IFN-γ in the endothelial cells of the portal veins and hepatic arteries may be implicated in the maternal immunoregulation through blood circulation during early pregnancy in sheep.

## Conclusion

There was downregulation of IFN-γ, IL-2, IL-4, IL-6 and IL-10, but IL-5 was upregulated in the livers during early pregnancy. Furthermore, the IFN-γ protein was limited to the endothelial cells of the proper hepatic arteries and portal veins. Therefore, we suggested that early pregnancy exerted its effect on the liver to regulate Th cytokines profile, but there was no evident shift from Th1 to Th2 cytokines, which may be necessary for the maternal hepatic immune adjustments during early pregnancy in sheep.
